# Dengue viruses binding proteins from *Aedes aegypti *and *Aedes polynesiensis *salivary glands

**DOI:** 10.1186/1743-422X-6-35

**Published:** 2009-03-25

**Authors:** Van-Mai Cao-Lormeau

**Affiliations:** 1 Head, Laboratoire de Recherche en Virologie Médicale, Institut Louis Malardé, Po Box 30, 98 713 Papeete, Tahiti, French Polynesia

## Abstract

Dengue virus (DENV), the etiological agent of dengue fever, is transmitted to the human host during blood uptake by an infective mosquito. Infection of vector salivary glands and further injection of infectious saliva into the human host are key events of the DENV transmission cycle. However, the molecular mechanisms of DENV entry into the mosquito salivary glands have not been clearly identified. Otherwise, although it was demonstrated for other vector-transmitted pathogens that insect salivary components may interact with host immune agents and impact the establishment of infection, the role of mosquito saliva on DENV infection in human has been only poorly documented. To identify salivary gland molecules which might interact with DENV at these key steps of transmission cycle, we investigated the presence of proteins able to bind DENV in salivary gland extracts (SGE) from two mosquito species. Using virus overlay protein binding assay, we detected several proteins able to bind DENV in SGE from *Aedes aegypti *(L.) and *Aedes polynesiensis *(Marks). The present findings pave the way for the identification of proteins mediating DENV attachment or entry into mosquito salivary glands, and of saliva-secreted proteins those might be bound to the virus at the earliest step of human infection. The present findings might contribute to the identification of new targets for anti-dengue strategies.

## Findings

As the third millennium begins, classic dengue fever and the more severe dengue hemorrhagic fever and dengue shock syndrome, are still world public health concerns. Every year, dengue virus (DENV) infects more than 50 million people, with approximately 22 000 fatal cases [1]. There are four antigenically distinct, but related, serotypes of DENV, a *Flavivirus *member of the family *Flaviviridae*. There is currently no vaccine available against DENV and vector control strategies fail to prevent the emergence of dengue epidemics, therefore new anti-dengue strategies need to be explored. A better understanding of the mechanisms and the molecules involved in the key steps of the DENV transmission cycle may lead to the identification of new anti-dengue targets.

DENV is transmitted by *Aedes (Stegomyia) *mosquitoes, principally *Ae aegypti *but also *Ae albopictus *and some endemic vectors like *Ae polynesiensis *in French Polynesia [2-4]. Infection of the female mosquito occurs during a blood feeding on a viremic human host. During the ten days following the ingestion of the infectious blood meal, viral replication occurs in different mosquito tissues and the virus finally infects the salivary glands [5-7]. Infection of mosquito salivary glands and subsequent injection of infectious saliva into the human host are key events of DENV transmission cycle.

In the present study, we investigated the presence of proteins able to bind to DENV in salivary gland extracts (SGE) from the *Ae aegypti *Bora-Bora strain (provided by the IRD, Montpellier, France) and an *Ae polynesiensis *wild colony from Atimaono-Tahiti (reared in our laboratory since 2000).

The salivary glands from 3–15 day-old adult females were dissected in phosphate buffer saline (PBS) 20 mM and immediately transferred into a vial containing a lysis buffer (1.5 mM MgCl_2_, 10 mM Tris-HCl, 10 mM NaCl, and 1% Nonidet P-40) and protease inhibitors (2 mM EDTA, 0.5 mM phenylmethylsulfonyl fluoride and 10 μg/ml of aprotinine). Each vial contained about 1,500 pairs of salivary glands and was stored at -80°C until needed [8]. Salivary glands were then thawed and disrupted by sonication in an ice-water bath before being centrifugated at 9,000 × *g *for 15 minutes at 4°C. The supernatant containing SGE was recovered for protein quantification and stored at -80°C [9]. To prepare semi-purified virus, the four reference strains of DENV (type 1, [*Hawaii*, Hawaii 1944]; type 2, [*New Guinea C*, Hawaii 1944]; type 3, [*H-87*, Philippines 1956]; type 4, [*H-241*, Philippines 1956]) and a clinical isolate obtained during the 1979 DEN4 epidemic in French Polynesia (amplified two times on *Ae albopictus *C6/36 cell cultures and stored at -80°C), were inoculated into the brain of suckling mice [10]. Mouse brain viral antigen extracts were then clarified by centrifugation at 12,000 × *g *for 5 minutes and supernatants were applied into a discontinuous gradient of 65% and 15% (w/w) sucrose in GNTE buffer (200 mM Glycine, 100 mM NaCl, 50 mM Tris-HCl, 1 mM Ethylene diamine tetracetate [EDTA]). Sucrose gradients were centrifuged at 21,500 × *g *for 3.5 hours at 4°C. The visible band containing the viruses was removed, diluted with GNTE and pelleted by centrifugation at 16,500 × *g *for 2 hours at 4°C. Finally the viral pellet was resuspended in GNTE and stored at -80°C [11,12]. For Virus Overlay Protein Binding Assay (VOPBA) total proteins from SGE were separated by SDS-10% polyacrylamide gel electrophoresis (PAGE), in non reducing conditions, before being transferred onto a nitrocellulose membrane [13]. Membrane sheets (one lane per sheet) were then incubated in PBS-5% (w/v) skim milk overnight at 4°C. The membranes were then blocked in PBS-0.5% (w/v) Tween20-5% skim milk for 1 hour at 37°C, followed by a first wash with PBS-0.5% Tween20 for 5 minutes followed by an additional wash with a high-salt wash-buffer (PBS, 0.5% Tween20, 1% skim milk, 220 mM NaCl). Membranes were then incubated for 3 hours at 37°C with DENV antigen extracts diluted in high-salt wash-buffer to obtain a final titre of 7.3 Log_10 _TCID_50_/ml. Virus binding was then detected indirectly by incubating nitrocellulose membranes with either anti-DENV type-specific hyperimmune mouse ascitic fluid (HMAF) or anti-envelop (E) protein type-specific monoclonal antibodies (Mabs). After incubation with horseradish peroxidase conjugated sheep anti-mouse IgG, DENV binding was visualized on X-ray film using an enhanced chemiluminescent (ECL) substrate.

**Figure 1:**
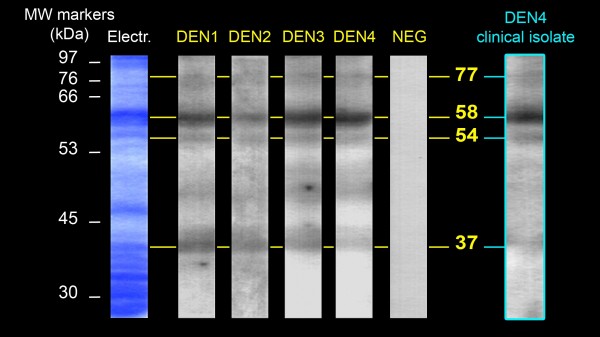
**DENV-binding proteins from Ae aegypti salivary glands detected with anti-DENV polyclonal antibodies**. Total proteins from *Ae aegypti *salivary gland extracts were separated on a SDS-PAGE (Electr) and transferred onto a nitrocellulose membrane. Membrane sheets were then incubated with either: DENV reference strains (DEN1 to DEN4); a semi-purified non-inoculated suckling mouse brain extract (NEG); or a DEN4 clinical isolate. Virus binding was detected using anti-DENV HMAF. Migration of the molecular weight markers and the estimated size of the DENV-binding proteins are indicated in kilodaltons (kDa), respectively on the left and on the right side of the figure.

**Figure 2:**
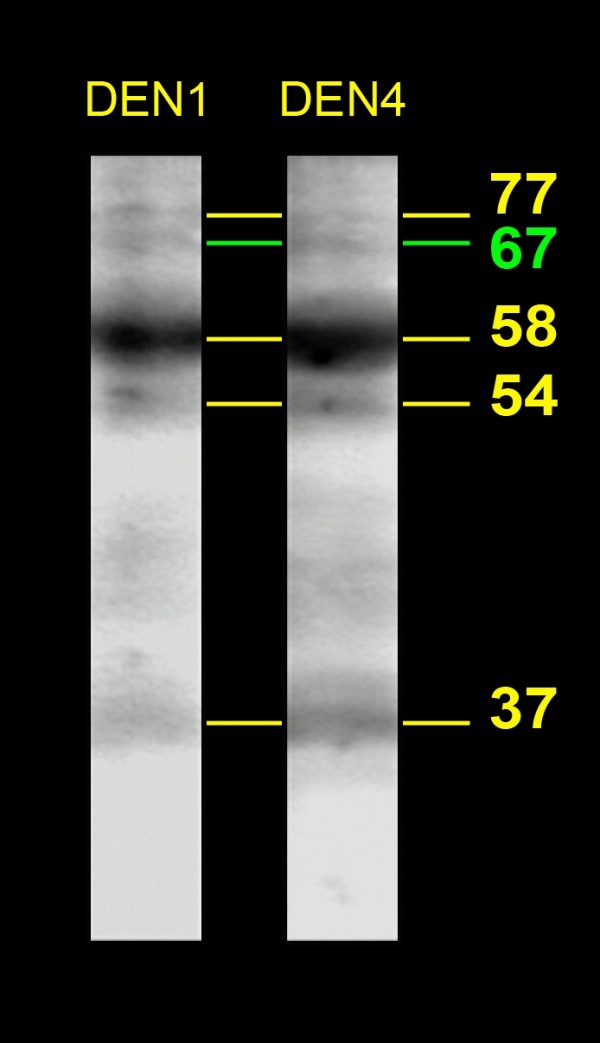
**DENV-binding proteins from *Ae aegypti *salivary glands detected with anti-E monoclonal antibodies**. Total proteins from *Ae aegypti *salivary gland extracts were treated as described in Figure 1. After transfer, membrane sheets were incubated with either: DEN1 or DEN4 reference strains. Virus binding was then detected using anti-E Mabs. The estimated size of the DENV-binding proteins are indicated in kilodaltons (kDa) on the right side of the figure.

**Figure 3:**
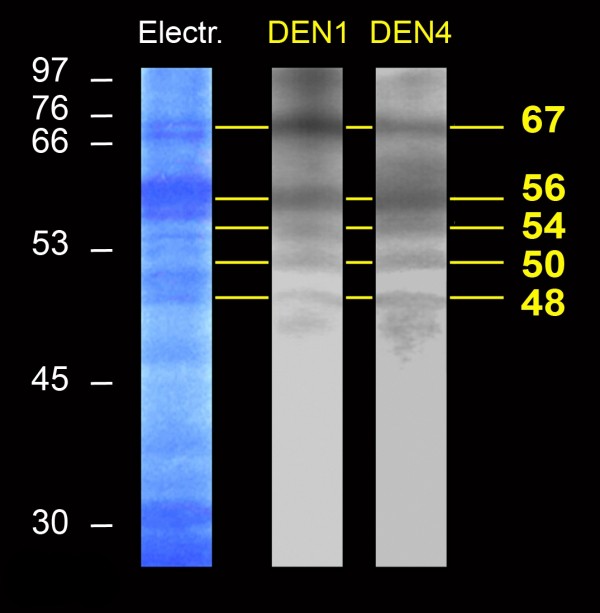
**DENV-binding proteins from *Ae polynesiensis *salivary glands detected with anti-E monoclonal antibodies**. *Ae *polynesiensis salivary gland extracts were treated as described for *Ae aegypti *in Figures 1 and 2. After the membrane sheets had been incubated with either the DEN1 or DEN4 reference strains, DENV binding was detected using anti-E type specific Mabs. Migration of the molecular weight markers and the estimated size of the DENV-binding proteins are indicated in kilodaltons (kDa), respectively on the left and on the right side of the figure.

VOPBA experiments were first performed using HMAF. Four proteins of 77, 58, 54 and 37 kilodaltons (kDa) able to bind to the reference strains of the four DENV serotypes were detected in SGE from *Ae aegypti *(Figure 1). Because the DENV reference strains had been maintained and passaged for many years in laboratories, we also performed the experiment using a DEN4 clinical isolate. All of the proteins previously detected with the reference strains also appeared with the clinical isolate. VOPBA experiments were then performed using anti-E DEN1 or DEN4 specific Mabs. In SGE from *Ae aegypti*, the four proteins previously observed with HMAF and an additional protein of 67 kDa were detected (Figure 2). In SGE from *Ae polynesiensis*, five proteins of 67, 56, 54, 50 and 48 kDa, were able to bind to DEN1 and DEN4 reference strains (Figure 3).

This is the first report on the presence of proteins able to bind to the four DENV serotypes in mosquito salivary gland extracts. Because SGE might contain both salivary gland tissue (*basal lamina *or salivary gland epithelial cells) and saliva-secreted proteins, the present work initiates the identification of either proteins mediating DENV infection of mosquito salivary glands or proteins bound to the virus at the early step of human infection.

DENV dissemination into mosquito tissues is dependant on the ability of the virus to penetrate several barriers: the midgut infection barrier (MIB), the midgut escape barrier (MEB) and the salivary glands. However, little is known about the mechanism and the molecules that allow the virus to pass these barriers. Most of the studies designed to identify putative DENV-receptors in mosquito tissues have been performed using the *Ae albopictus *C6/36 cell line model [14-16]. There are only few reports on the isolation of such receptors in whole mosquito tissues. Yazi Mendoza et al. (2002) first described the presence of a DEN4-binding protein in mosquito tissues (head, thorax and abdomen), more recently Mercado-Curiel et al. (2006) report the isolation of two DENV putative receptors in midgut extracts from *Ae aegypti *[12,17]. However, receptors for all DENV serotypes in mosquito salivary glands have never been formally identified. Therefore, the next step to the present work would be to characterise the proteins that might be involved in DENV infection of *Ae aegypti *and *Ae polynesiensis *salivary glands. Once identified, such receptors would constitute key targets for transmission blocking strategies still explored for other vector-transmitted pathogens. As an example, the use of antibodies directed to *Plasmodium *sporozoïtes-receptors prevented the invasion of mosquito salivary glands by the parasite [18,19].

The saliva of several mosquito species has been well studied for its anti-haemostatic properties allowing efficient blood feeding [20]. The presence of pharmacologically active molecules such as platelet aggregation inhibitors, vasodilator agents and anti-coagulants have been described for *Ae aegypti *saliva [8,21-24]. The effect of the saliva from pathogen-transmitting arthropods on mammal host immune response and the establishment of the pathogen has also been investigated. There are several reports on the tick or the sand fly saliva to enhance bacterial, viral or parasite infections [25]. However, there has been little study on the arbovirus/mosquito saliva pair [26,27]. Using an *in vivo *mouse model it was recently demonstrated that mosquito feeding or mosquito saliva potentiates West Nile virus infection [28]. When focusing on the DENV/*Ae aegypti *pair, the only report is an *in vitro *study on human dendritic cells showing an inhibitory effect of vector saliva on DENV infection [29]. Because of the absence of a reliable animal model, the impact of *Ae aegypti *saliva on DENV infection in the human host remains unknown. The identification of mosquito salivary proteins able to form complexes with DENV would lead to new hypotheses on the role of vector saliva on the establishment of viral infection. Such complexes would be a live illustration of the conceptual surface-mosaic model that address the potential significance of protein adsorption to the surface of micro-organisms at the early phase of host-pathogen relationships [30].

## Competing interests

The author declares that they have no competing interests.
